# Kinetics of MDR Transport in Tumor-Initiating Cells 

**DOI:** 10.1371/journal.pone.0079222

**Published:** 2013-11-01

**Authors:** Vasilij Koshkin, Burton B. Yang, Sergey N. Krylov

**Affiliations:** 1 Department of Chemistry and Centre for Research on Biomolecular Interactions, York University, Toronto, Ontario, Canada; 2 Sunnybrook Research Institute, Sunnybrook Health Sciences Centre, Toronto, Ontario, Canada; Technion-Israel Institute of Technology, Israel

## Abstract

Multidrug resistance (MDR) driven by ABC (ATP binding cassette) membrane transporters is one of the major causes of treatment failure in human malignancy. MDR capacity is thought to be unevenly distributed among tumor cells, with higher capacity residing in tumor-initiating cells (TIC) (though opposite finding are occasionally reported). Functional evidence for enhanced MDR of TICs was previously provided using a “side population” assay. This assay estimates MDR capacity by a single parameter - cell’s ability to retain fluorescent MDR substrate, so that cells with high MDR capacity (“side population”) demonstrate low substrate retention. In the present work MDR in TICs was investigated in greater detail using a kinetic approach, which monitors MDR efflux from single cells. Analysis of kinetic traces obtained allowed for the estimation of both the velocity (*V*_max_) and affinity (*K*_M_) of MDR transport in single cells. In this way it was shown that activation of MDR in TICs occurs in two ways: through the increase of *V*_max_ in one fraction of cells, and through decrease of *K*_M_ in another fraction. In addition, kinetic data showed that heterogeneity of MDR parameters in TICs significantly exceeds that of bulk cells. Potential consequences of these findings for chemotherapy are discussed.

##  Introduction

One of the most important aspects of tumor heterogeneity is the existence of tumor-initiating cells (TICs) responsible for both the initiation of cancers and dissemination of metastases [1,2]. Besides their elevated proliferative and invasive capacity, these cells also demonstrate high resistance to anticancer treatments, in particular, to chemotherapy (chemoresistance). One of the mechanisms behind chemoresistance is the overexpression of plasma membrane pumps (ABC transporters) which expel chemotherapeutics from the cell interior [3]. The overall functional characterization of MDR in TICs to date has been provided by a widely used flow cytometric side population assay. This assay is based on the differential accumulation of a fluorescent MDR substrate, Hoechst 33342, in cancer cells. Specifically, the cells exhibiting low Hoechst retention are considered as having high MDR capacity and referred to as the “side population”[4].

Intracellular dye retention is a complex parameter reflecting, on one hand, cellular dye uptake and, on the other hand, turnover and affinity of the dye efflux system. A more detailed quantitative characterization of MDR transport in TICs requires the determination of its kinetic transport parameters (*V*_max_ and *K*_M_) and transport efficiency (*V*_max_/*K*_M_). Such a study is of particular importance, since besides expressional regulation, MDR transporters are subject to functional modulation, for instance, by a transporter’s membrane environment and the cellular metabolic state. For example, the reported controversy on the relation between a cell’s tumor-initiating ability and its chemoresistance [5,6] might be caused by multifactorial regulation of MDR transport kinetics in different cell subpopulations. A detailed kinetic characterization of MDR function in TICs is increasingly important in light of the fact that elevated chemoresistance of TICs may result in the increase of TIC fraction and subsequent malignancy in the residual cancer after chemotherapy [7,8].

The aim of this work was the kinetic characterization of MDR in TICs in comparison to MDR in bulk tumor cells. As an experimental object of this work we used the intrinsic MDR capacity of naïve (not exposed to MDR-inducing therapeutics) MCF-7 and 4T1 breast cancer cells. This type of MDR reflects cell/drug interaction upon the first clinical application of cancer chemotherapy.

The relatively high number of TICs required for this work was generated using the tumorpshere approach based on anchorage-independent cell growth. This kind of cancer cell growth is known to favour the proliferation of TICs compared to regular monolayer growth [9]. MDR transport in TICs and bulk cells was estimated at the single cell level and characterized by the *V*_max_ and *K*_M_ parameters (we [10] and others [11] have previously shown a significant advantage of single-cell measurements over population-based measurements in kinetic studies). We found that activation of MDR transport in TICs, compared to transport in bulk cells, is highly heterogeneous and realized by two alternative mechanisms. These mechanisms are an elevation of MDR activity (*V*_max_) in one subpopulation of TICs and an increase of MDR affinity (decrease of *K*_M_) in another subpopulation. Potential consequences of these findings for cancer chemotherapy are discussed. 

## Materials and Methods

### 1. Cell and tumorsphere culture conditions

MCF-7 human and 4T1 murine breast cancer cells were purchased from the American Type Culture Collection (ATCC, Manassas, VA, USA) and grown in monolayers as recommended by ATTC. To generate tumorspheres, the floating (loosely adherent) cells were collected by washing ~70% confluent culture dishes with growth medium [12]. Collected floating cells were then cultured in ultralow attachment plates (Corning, Acton, MA, USA) under serum-free conditions using medium # 05621 with appropriate supplements developed by STEMCELL Technologies (Vancouver, BC, Canada).

### 2. Tumorsphere formation efficiency assay

Trypsinized monolayers and tumorspheres were plated onto a 96-well plate (200 cells/well, medium # 05621, STEMCELL Technologies, Vancouver), the number of spheres formed was counted in 5 days [9].

### 3. Cell chemoresistance

Cell chemoresistance was deduced from cell viability in the presence of an anticancer agent, that was determined using a standard colorimetric MTT (3-4,5-dimethylthiazol-2-yl-2, 5-diphenyl-tetrazolium bromide) reduction assay [13]. As previously described [14], after a period of growth in 96-well plates (48 h at 37°C, 10,000 cells/well, with or without 30 nM doxorubicin), cells were supplemented with MTT (0.5 mg/mL) and incubated for 4 h. Redox activity in viable cells converted the oxidized form of MTT into the reduced formazan form. Formazan crystals were released from cells and dissolved using SDS (3% final content). Redox activity/cell viability was estimated from formazan absorbance measured at 550 nm and expressed as percentage of absorbance for the untreated control.

### 4. Measurement of MDR transport in single cells by fluorescence kinetic microscopy

MCF-7 cells grown to 50–60% confluence were supplemented with 10 µM glyburide (MDR inhibitor) and loaded with 5 µM fluorescein (MDR substrate) for 30 min at 37°C (with 4T1 cells calcein-AM was used as MDR substrate). The cells were then washed free of extracellular fluorescein and glyburide and placed in the KRB buffer (115 mM NaCl, 5.9 mM KCl, 2.5 mM CaCl_2_,1.2 mM MgCl_2_, 1.2 mM NaH_2_PO_4_, 15 mM NaHCO_3_, 10 mM glucose, pH 7.4). The kinetics of fluorescein efflux were monitored with kinetic fluorescent microscopy and analysed by means of Cell Profiler (Broad Institute, Cambridge, MA) [15] and Origin (Microcal, Northampton, MA) software following an approach described in detail elsewhere [10,16]. Control for variation of cell volume during time-lapse measurement was performed using z-stacks taken before and after time-lapse acquisition. Stacks were analysed with FluoView version 5 (Olympus, Tokyo, Japan) and ImageJ (NIH, Bethesda, MD) [17] software packages.

### 5. Assessment of CD44/CD24 status of the cells

After completing the MDR efflux assay, the cells were subjected to immunophenotyping (assessing quantity of CD44 and CD24 biomarkers) for discrimination between TICs and bulk cells within the cell population in a field of view. Immunostaining and imaging were based on the protocol described by Gupta et al. [18], except that FITC and PE/Cy5 were used as fluorescent labels instead of Alexa Fluo 488 and Alexa Fluo 555, respectively. Fluorescence images of fixed and antibody-treated cells were captured (with a fully open confocal aperture) using a confocal laser scanner FV300 (Olympus, Tokyo, Japan) at 530 and 665 nm. Between 4 to 9 scans were performed and averaged to improve the signal to noise ratio of the final image. Cell fluorescence intensities were quantitated, presented as 2-D plots and subjected to image-based cytometric analysis for separation of bulk and TIC subpopulations. To establish gates on the plots determining subpopulations characterized by high and low marker expression, we used the well known fact that an MCF-7 cell monolayer culture has null or negligible content of TICs [19,20].

### 6. Toluidine blue cell staining

Toluidine blue cell staining, used as an alternative cell sorting criterion, was performed according to a previously published protocol [12,21]. The staining intensity was estimated using an FV300 laser scanner in transmission mode.

### 7. Flow cytometric measurements of MRP1 expression and plasma membrane permeability

MRP1 expression was estimated using flow cytometry with FITC mouse anti-human MRP1 antibody QCRL-3 and appropriate isotype control (BD Biosciences, San Jose, CA, cat # 557593 and 555573, respectively) according to published procedure [22]. Results were analyzed ratiometrically as mean fluorescence with MRP1-specific antibody divided by mean fluorescence with isotype control [23]. Plasma membrane permeability was tested using two-color flow cytometric assay which estimates a cell’s ability to retain fluorescein derivative bis-carboxyethyl carboxy fluorescein (BCECF) and to exclude propidium iodide (PI) [24]. Measurements were performed using BD FACSCanto II Flow Cytometer. 

## Results

### 1. Tumorigenic and chemoresistant properties of monolayer and tumorsphere cells

First, we assessed tumorigenic potential (using its *in vitro* criterion anchorage-independent growth [25]) and chemoresistance of total cell populations derived from MCF-7 monolayers and tumorspheres. This was performed by determining the efficiency of tumorsphere formation and survival rate in the presence of an anticancer agent in both types of cells. 

**Figure 1:**
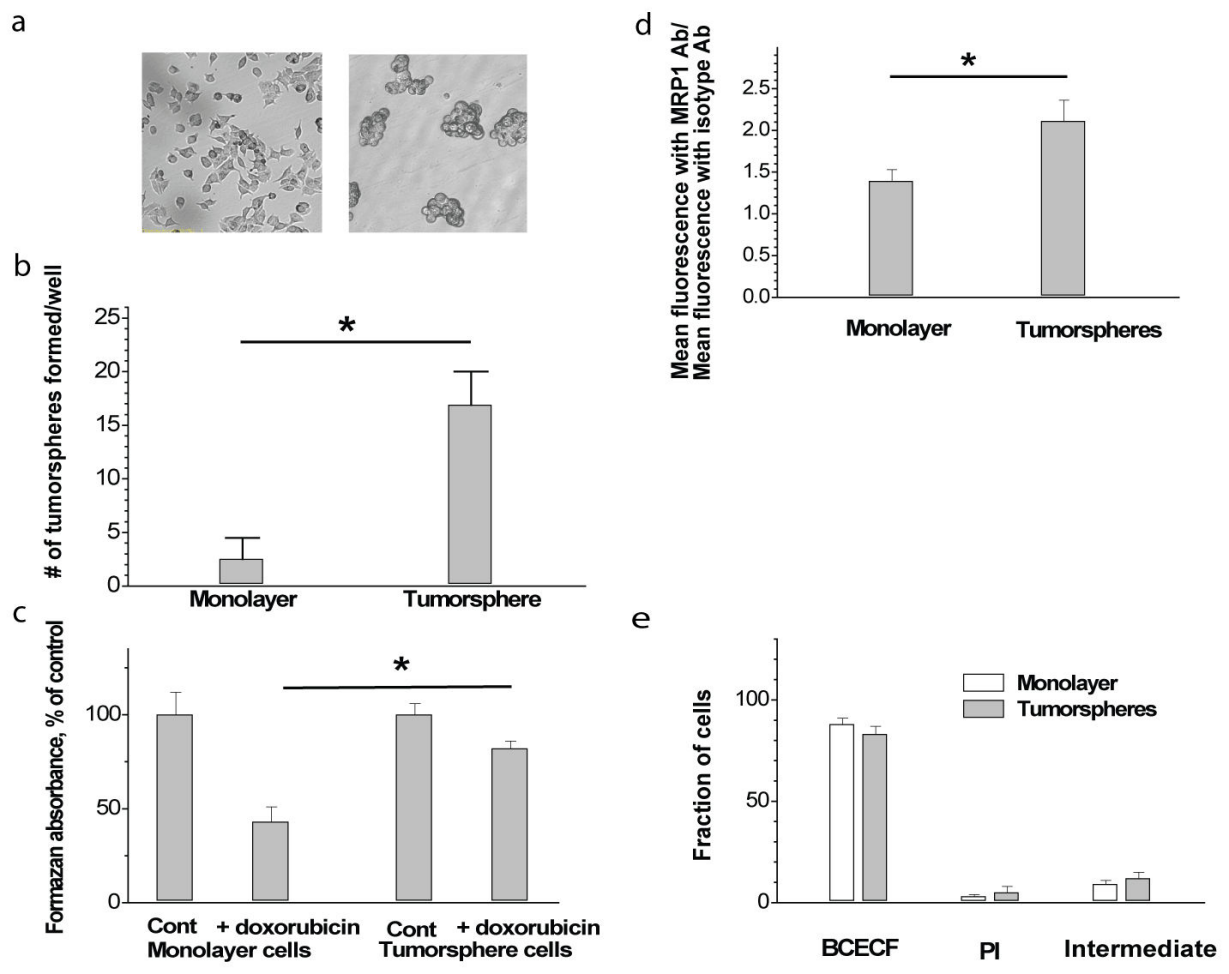
Cells grown in monolayers (a, left image) and tumorspheres (a, right image) show different ability to form secondary tumorspheres (b), resistance to doxorubicin (c), expression of MRP1 (d), and similar ability to retain BCECF and exclude PI (e). Bars represent means ± SE, *p < 0.05.

Monolayers (Figure 1a, left image) and tumorspheres (Figure 1a, right image) were dissociated and subjected to tumorsphere formation efficiency assay (see Materials and methods). Figure 1b indicates that cells originating from tumorspheres showed a significantly higher ability to undergo anchorage-independent growth compared to monolayer cells (*p* < 0.05, *n* = 3). 

Overall estimation of chemoresistance in these types of cells was performed by comparison of the chemotoxic effect exerted by a low dose of doxorubicin on cells from dissociated tumorspheres and regular monolayers. Results shown in Figure 1c indicate better survival of the tumorsphere-derived cells compared to cells from monolayers in the presence of low dose doxorubicin (*p* < 0.05, *n* = 3). 

Obviously improved survival of tumorsphere cells can be tentatively attributed to their increased multidrug resistance. We [10] and others [14,26] have previously shown that MCF-7 cells possess moderate intrinsic multidrug resistance of MRP-type. We therefore compared the expression of the MRP1, the most commonly encountered transporter of this type, in monolayers and tumorspheres. Comparative investigation showed somewhat higher MRP1 expression level in tumorspheres (Figure 1, d). However, MDR function is determined by many factors besides transporter expression, and poor and even negative correlation between expression and function of MDR transporters is not uncommon [27,28]. Therefore, functional evidence was required to understand the mechanisms of elevated chemoresistance in TICs. 

As a preliminary precaution, we tested both cell types for plasma membrane permeability which might contribute to different transmembrane drug distribution in these cells. We applied the widely used analysis of plasma membrane permeability with complementary fluorescent probes [24], which assesses membrane permeability in terms of cell fractions showing dye retention (with the dye BCECF), dye exclusion (with PI dye) and intermediate fraction. Figure 1, e shows that these parameters in tumorspheres remain close to those in monolayer cells. 

### 2. MDR efflux and estimation of functionally active MDR transporter content in naïve MCF-7 cells

**Figure 2:**
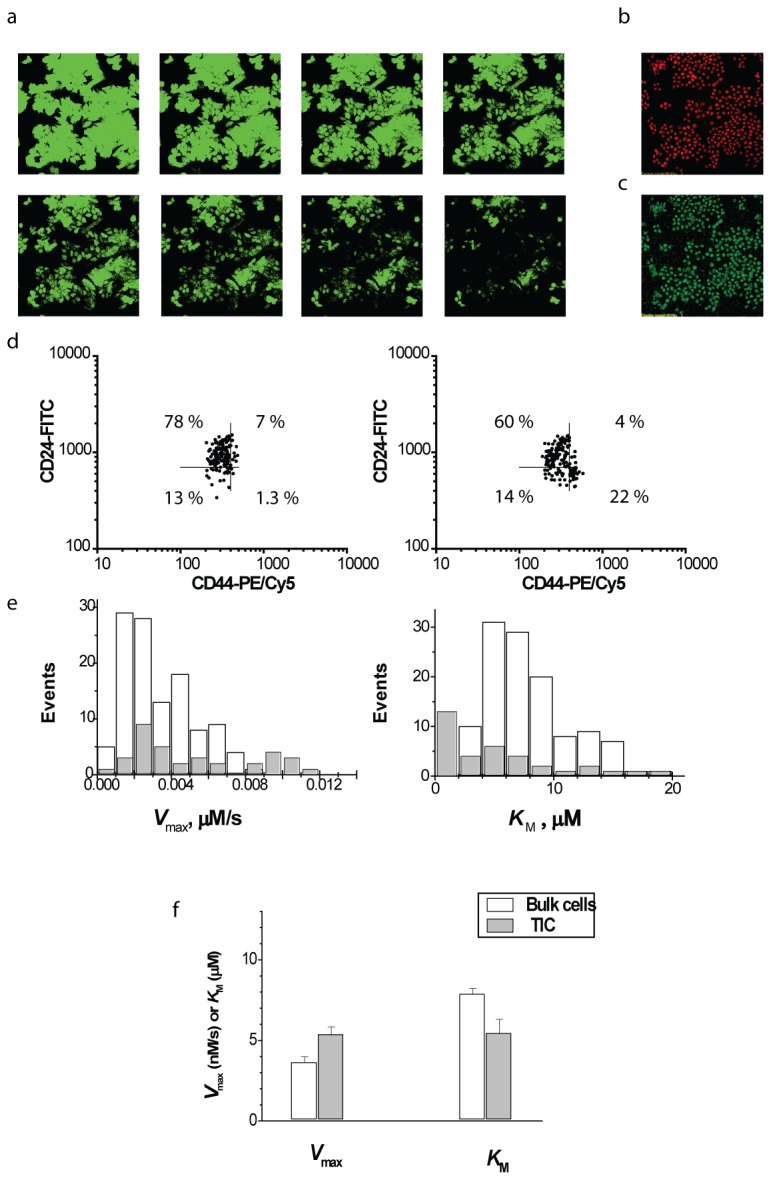
Kinetic and immunophenotyping analysis of MCF-7 TICs and bulk cells: (a) images taken at 10 min intervals illustrate MDR efflux from cells loaded with fluorescein; (b) immunostaining of the same cells for CD44; (c) immunostaining of the same cells for CD24; (d) 2-D plots of the fluorescence intensity of CD44 and CD24 signals in monolayer- ( left) and tumorsphere-derived cells (right); (e) histograms of distribution of Michaelis parameters within CD44^high^/CD24^low^ cells (gray bars) and bulk cells (white bars) demonstrating two ways of MDR activation in TICs; (f) cumulative data summarizing Michaelis parameters in CD44^high^/CD24^low^ cells (gray bars) and bulk cells (white bars) in 5 independent **experiments **(**total 1089 cells, *p* < 0.01)**.

Here we examined MDR efflux in MCF-7 cells belonging to the TIC or bulk subpopulations using fluorescence kinetic imaging. To allow for the possible photoinduced cell shrinkage, we tested stability of cell volumes in the course of time-lapse cell observation (see Materials and methods). It was determined that variation of cell volume during time series acquisition is below experimental error in cell volume estimation (9 %). The cells were loaded with fluorescein (substrate of MRP family of MDR transporters) and allowed to extrude it. The extrusion process was monitored by scanning fluorescent microscopy (Figure 2a), and kinetic description of MDR transport in single cells was derived as described in detail previously [10,16]. Naturally, this intrinsic activity is significantly lower than the induced activities of MRP and other types of transporters observed in cells exposed to different drugs [10]. For a rough estimate of the number of MDR transporter molecules driving this activity we used the average maximum rate of fluorescein efflux determined in this and previous work [10] *V*_max_ ≈ 3 nM/s and a turnover number typical for ABC transporters [29] *TN* ≈ 10 s^-1^. Since *V*_max_ = TN × [enzyme]_total_, comparison of these values showed that the [enzyme]_total_ in this system is about 0.3 nM. Assuming the cell volume to be ~ 2 pL [30] we estimated the cellular enzyme content to be below 1,000 molecules per cell. At such a low target level, accuracy of immunostaining can be compromised [27,31], which heightens the importance of functional MDR investigation. 

### 3. MDR transport in MCF-7 tumorsphere-forming cells sorted by cell surface biomarker criterion

Breast cancer tumorspheres (mammospheres) are known to contain a significant fraction of TICs [9,32]. This property of tumorspheres allows us to get TIC-related information from monitoring MDR efflux in the whole cell population produced by dissociation of tumorspheres. After the completion of kinetic measurements, the cells were sorted into TIC and bulk cell subpopulations according to the expression levels of commonly used biomarkers CD44 and CD24. In order to combine kinetic and immunophenotype descriptions of individual cells we used microscopic (imaging) immunophenotyping instead of flow cytometry. This technique is finding increasing application in situations where flow cytometry is not sufficient [33-35]. Repetitive scanning of the cells immunostained after completion of the MDR assay (see Materials and methods for details) was followed by scan averaging for signal- to-noise ratio improvement. Resulting images (Figures 2b and 2c) closely resemble reported images of cells immunostained without a preceding MDR assay [18,36], suggesting no significant interference between the MDR assay and immunostaining. Fluorescence intensities of individual cells were organized and displayed as a 2D plot (Figure 2d). Considering the lower right quadrant of the gated 2D plot (Figure 2d) as the TIC fraction, we compared frequency distributions of single-cell *V*_max_ and *K*_M_ values from this quadrant with the rest of the diagram (Figure 2e). It is clear that the CD44^high^/CD24^low^ subpopulation is characterized by the right shift in *V*_max_ distribution and left shift in the distribution of *K*_M_. In fact, the distribution of *V*_max_ suggests the separation of the fraction of CD44^high^/CD24^low^ cells having elevated *V*_max_ from the cells demonstrating slow transport. In the *K*_M_ distribution within CD44^high^/CD24^low^ cells separation of cell fraction with high affinity from low affinity cells is less pronounced. However, the most populated (modal) bin in the CD44^high^/CD24^low^ histogram was always the lowest one. In the bulk cell histograms the maximal bin was distant from the histogram edge. It is possible that the downward shift in the *K*_M_ distribution was partially masked by the general left skewed shape of these distributions. Interestingly, the alterations in *V*_max_ and *K*_M_ did not correlate with each other, thereby suggesting the existence within the whole CD44^high^/CD24^low^ subpopulation of three types of cells: cells with decreased *K*_M_ , cells with elevated *V*_max_ , and cells remaining similar to those in the bulk tumor. A statistical summary of kinetic parameters characterizing TICs and bulk cells is shown in Figure 2, f. 

**Figure 3:**
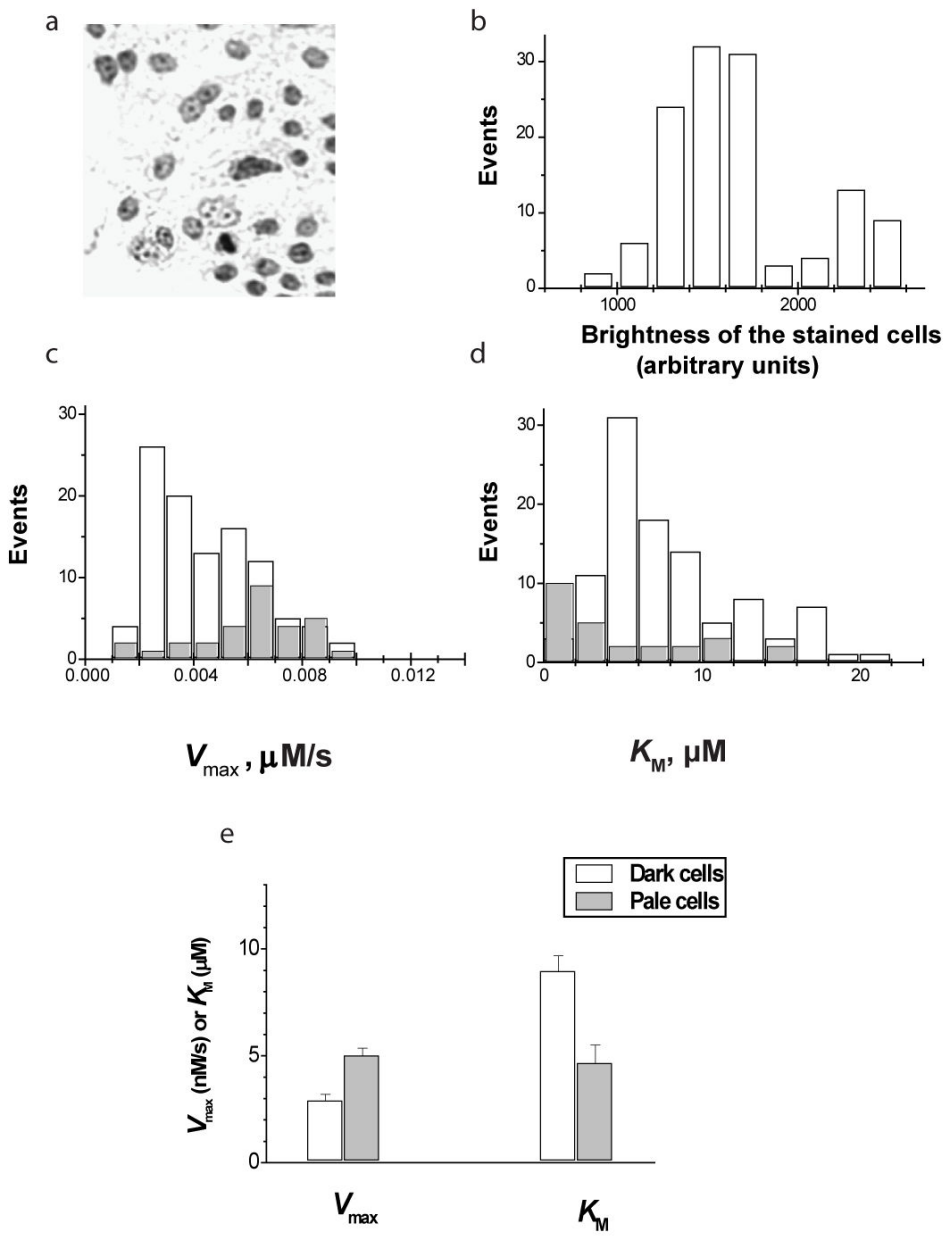
Kinetic and morphologic analysis of MCF-7 TICs and bulk cells: (a) typical image of toluidine blue-stained cells; (b) distribution of brightness of stained cells showing discrimination between pale and dark cells; (c, d) histograms of distribution of Michaelis parameters within pale (undifferentiated) cells (gray bars) and dark (differentiated) cells (white bars) demonstrating two ways of MDR activation in TICs; (e) cumulative data summarizing Michaelis parameters in pale (undifferentiated) cells (gray bars) and dark (differentiated) cells (white bars) in 4 independent experiments (** total 821 cells, *p* < 0.01)**.

We were also interested in determining the degree of *V*_max_ and *K*_M_ dispersion within TIC and bulk cell populations. Distributions of *V*_max_ and *K*_M_ parameters within these populations considerably deviate from normal (Figure 2, d, e; Figure 3, c, d), therefore we chose to characterize *V*_max_ and *K*_M_ dispersion using the robust coefficient of variation (rCV). rCV is the ratio of interquartile range which spans the central 50% of a data set, to the median value of a data set, which is more appropriate than common CV for description of skewed distributions [37]. Calculations showed that rCVs of *V*_max_ and *K*_M_ distributions of the TIC population were approximately 2-fold higher than those of the bulk population (data not shown). 

### 4. MDR transport in MCF-7 tumorsphere-forming cells sorted by cell morphology criterion

Next we confirmed the acceleration of MDR kinetics in TICs using intensity of cellular staining with toluidine blue as an independent cell sorting criterion. Toluidine blue is a cationic dye whose intensity of staining reflects the degree of differentiation of mammary epithelial cells, so that pale staining corresponds to less differentiated cells (presumably stem cells and early progenitors) [12,21]. Figure 3,e shows that *V*_max_ and *K*_M_ of the bright (differentiated) and pale (undifferentiated) cells differ in such a way that undifferentiated cells demonstrate a higher rate and tighter affinity of MDR transport. However, using toluidine blue-based cell sorting we observed a general upward shift of the *V*_max_ distribution in pale cells rather than separation between fast and slow subpopulations.

### 5. MDR transport in 4T1 cell subpopulations tumorsphere-forming cells sorted by cell surface biomarker criterion

We examined the generality of kinetic MDR features found in TICs of MCF-7 cell line by studying MDR efflux in murine breast cancer cell line 4T1. This type of cells also forms tumorspheres with elevated expression of CD44^high^/CD24^low^ phenotype [38] but shows mixed multidrug resistance of MDR and MRP types [39,40]. Therefore the kinetics of MDR efflux was measured with universal MDR substrate calcein [41] using cell array slides (Molecular Cytomics Inc., Boston, MA [42]), otherwise experimental protocol was similar to that used with MCF-7 cells.

**Figure 4:**
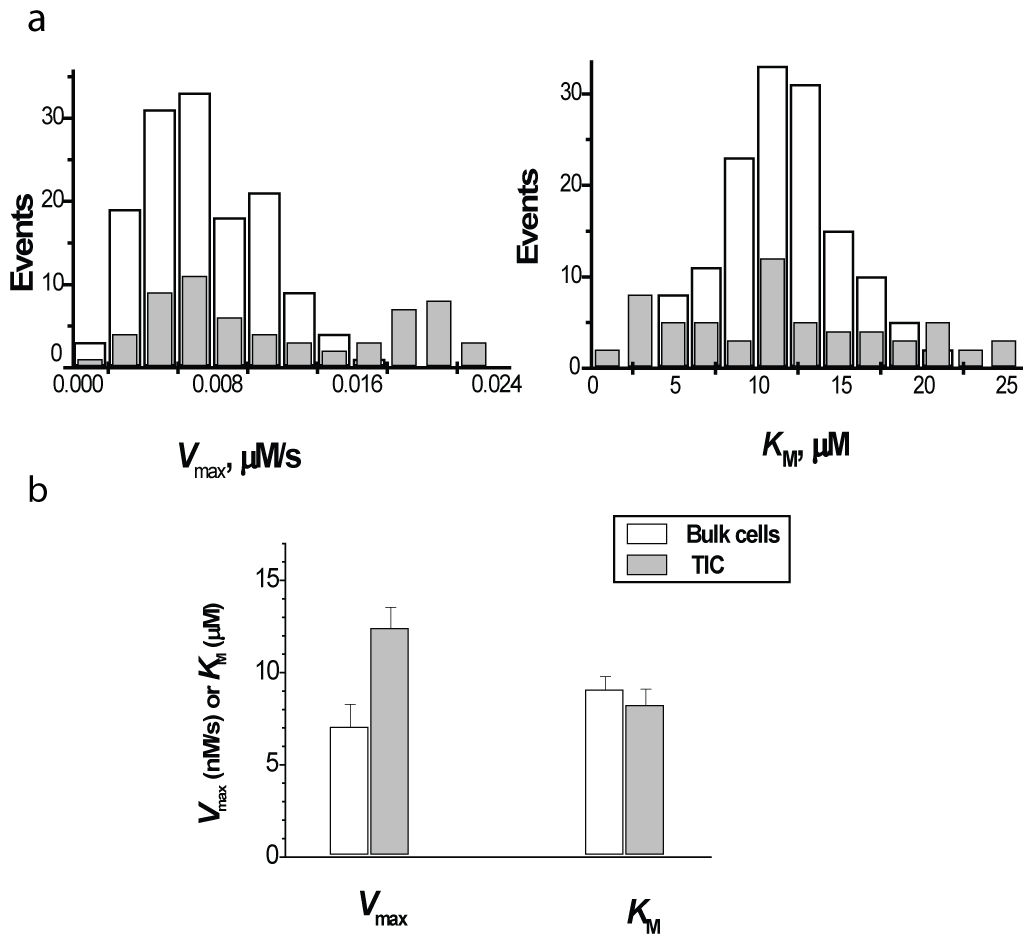
Kinetic and immunophenotyping analysis of 4T1 TICs and bulk cells: (a) histograms of distribution of Michaelis parameters within CD44^high^/CD24^low^ cells (gray bars) and bulk cells (white bars); (b) cumulative data summarizing Michaelis parameters in CD44^high^/CD24^low^ cells (gray bars) and bulk cells (white bars) in 4 independent experiments (total 709 cells, *p* < 0.02 for *V*_max_).

Distribution of *V*_max_ and *K*_M_ values within bulk cells and TICs, as well as cumulative mean data are shown in Figure 4. Similarly to MCF-7 cells, TICs in 4T1 cell line show wider than bulk cells distribution of *V*_max_ values with increased mean value. Range of *K*_M_ variation in TICs is also broader than in bulk cells, but, in contrast to MCF-7 cells, *K*_M_ mean values in both subpopulations of 4T1 cells are close to each other. However, due to broadened variation range TIC in 4T1 line, like in MCF-7 line, contain a significant fraction of cells with increased affinity (reduced *K*_M_). Thus, data on two cancer cell types suggest that MDR kinetics in TIC differ from these in bulk cells by: (i) broader variation of *V*_max_ and *K*_M_ parameters, (ii) increased *V*_max_ mean value, (iii) formation within TIC of a fraction of cells with elevated MDR affinity (reduced *K*_M_). 

Moreover, an important feature of TICs is diminished proportion of cells in G2/M phase due to slower progression of cell cycle [43,44]. We found recently that G2/M cells have greater *V*_max_ values of MDR flux compared to G1 cells [10]. Thus different rates of cell cycle in TICs and bulk cells can make observed difference in *V*_max_ smaller than it actually is.

## Discussion

It has been previously shown that the fraction of CD44^high^/CD24^low^ cells in tumorspheres is significantly elevated compared to classical monolayer cell cultures [32,45,46]. This fact solves the technical problems associated with rare cell studies [47]. Using cell preparations derived from tumorspheres allowed us to extract TIC single-cell kinetics of MDR transport from moderately large cell populations.

These results provide quantitative kinetic evidence supporting the concept of elevated MDR in TICs. Moreover, the application of the single-cell kinetics approach produces a more comprehensive description of the TIC chemoresistance capacity. It shows the MDR-related heterogeneity of TICs (CD44^high^/CD24^low^ subpopulation) which consist of 3 types of cells: cells with elevated rate of MDR efflux, cells with elevated MDR affinity, and cells with bulk cell MDR parameters. It is apparent that modulation of MDR transport in TICs caused by expressional and functional alterations in membrane transporters involves catalytic and binding effects [48]. 

According to a theoretical treatment of transmembrane drug equilibration, the kinetic definition of the degree of drug resistance (for low drug levels, typical for *in vivo* conditions) is given by: *R* = 1 - (*V*_max_/(*PK*_M_)), where *P* is drug permeability [49]. Thus both *V*_max_ increase and *K*_M_ decrease of the MDR transport improve a TIC’s drug resistance. At the same time, one can expect that changes in *V*_max_ have more general character than changes in *K*_M_. While altered *V*_max_ (*V*_max_ = *k*_cat_ ×[transporter]) relates to a particular transporter, altered *K*_M_ relates to a particular transporter/substrate combination and may change as the substrate changes. For instance, modulation of plasma membrane properties affects the affinity of MDR transporter for some substrates, but not for others [50]. Thus it is conceivable, that in some cases reduced MDR affinity compensates for increased MDR activity. This kind of effect might explain recent reports arguing against MDR activation in TICs [5,6]. 

Increased variation (rCV) of *V*_max_ and *K*_M_ in TICs shows that heterogeneity in TICs significantly exceeds that in bulk tumor cells. Phenotypic heterogeneity within cancer cell populations attracts significant attention as a clinically important parameter [51,52]. There is strong evidence that this heterogeneity has a dynamic nature and involves interconversion between different phenotypes due to fluctuations in the levels of cell constituents [52]. As applied to TIC populations in this work, this concept implies interconversions between TICs with high and low MDR capacity. It was recently suggested that such sytems can be subjected to specific therapeutic approach [52]. According to this approach, drug doses intended for killing bulk cells can be efficient against TICs if drug application is synchronized with the kinetics of interconversion between TICs with high and low MDR. 

Occurrence of a fraction of TICs with increased MDR affinity (decreased *K*_M_) has not been reported previously, and clinically can be an additional mechanism for the positive selection of TICs during chemotherapy [7,8]. Preferential survival of TICs under chemotherapy is known to result from an overexpression of MDR transporters, i.e., increased MDR *V*_max_. Our data show that in a certain range of concentrations of chemotherapeutic (*K*_M TIC_ < [chemotherapeutic] < *K*_M bulk cell_), MDR transporters in TICs with improved affinity will be better saturated with substrate than those in bulk cells. Thus, even without expressional effects, MDR transport and chemoresistance in TICs will exceed those in bulk cells. This difference provides an additional mechanism for the increase of TIC content in tumors subjected to chemotherapy.

Classical “side population” assay performed at a standard substrate load [53] provides a functional estimation of MDR transport but does not determine its affinity. In contrast, monitoring of the full kinetics of MDR transport in single cells provides estimates of both velocity and affinity of the process. This information would be useful for the rational design of therapeutic regimens, especially for prevention of tumor TIC enrichment during chemotherapy.
